# How and why should we engage parents as co‐researchers in health research? A scoping review of current practices

**DOI:** 10.1111/hex.12490

**Published:** 2016-08-12

**Authors:** Shuoqi Shen, Krissy A.R. Doyle‐Thomas, Lori Beesley, Amir Karmali, Laura Williams, Nadia Tanel, Amy C. McPherson

**Affiliations:** ^1^ Bloorview Research Institute Toronto ON Canada; ^2^ Holland Bloorview Kids Rehabilitation Hospital Toronto ON Canada; ^3^ Health Quality Canada Toronto ON Canada; ^4^ University of Toronto Toronto ON Canada

**Keywords:** family‐centred care, participatory research, patient engagement, patient involvement

## Abstract

**Background:**

The importance of engaging parents in health research as co‐researchers is gaining growing recognition. While a number of benefits of involving parents as co‐researchers have been proposed, guidelines on exactly how effective engagement can be achieved are lacking. The objectives of this scoping review were to (i) synthesize current evidence on engaging parents as co‐researchers in health research; (ii) identify the potential benefits and challenges of engaging parent co‐researchers; and (iii) identify gaps in the literature.

**Methods:**

A scoping literature review was conducted using established methodology. Four research databases and one large grey literature database were searched, in addition to hand‐searching relevant journals. Articles meeting specific inclusion criteria were retrieved and data extracted. Common characteristics were identified and summarized.

**Results:**

Ten articles were included in the review, assessed as having low‐to‐moderate quality. Parent co‐researchers were engaged in the planning, design, data collection, analysis and dissemination aspects of research. Structural enablers included reimbursement and childcare. Benefits of engaging parent co‐researchers included enhancing the relevance of research to the target population, maximizing research participation and parent empowerment. Challenges included resource usage, wide‐ranging experiences, lack of role clarity and power differences between parent co‐researchers and researchers. Evaluation of parent co‐researcher engagement was heterogeneous and lacked rigour.

**Conclusions:**

A robust evidence base is currently lacking in how to effectively engage parent co‐researchers. However, the review offers some insights into specific components that may form the basis of future research to inform the development of best practice guidelines.

## 1 Introduction

Client and family‐centred care (CFCC) highlights the family as being integral to children's well‐being and is now widely recognized as essential to the field of child health.^1^, ^2^, ^3^ CFCC as it relates to child health has historically been rooted in the principals of partnership and collaboration between parents and professionals.^1^, ^2^, ^4^ It recognizes that family members are usually the constant in a child's life and as such represent considerable expertise in the child's health and care.^2^, ^3^, ^4^, ^5^

The concept of engaging patients and their families in health *research* greatly aligns with the principals and core beliefs of CFCC and has also recently gained ground and attracted the attention of researchers and policymakers.^6^, ^7^, ^8^, ^9^ Government funding agencies have created specific mechanisms through which to promote patient engagement in research, such as the Strategy for Patient‐Oriented Research initiative in Canada and the Food Drug Administration Patient Engagement Advisory Committee in the United States. However, while systematic reviews have focused upon involving patients and their families in research generally,^8^, ^10^ none have looked at parents specifically. While it is vital to engage well children in research, parents can represent those who may not themselves be able to act as co‐researchers, such as those with severe disabilities or young children. As such, parents play a key role in fields such as paediatric long‐term illness and rehabilitation, in addition to health promotion and public health‐related areas.^5^ Parenting children with on‐going health‐care concerns presents unique challenges typically not faced by other populations due to the long‐term nature of the conditions, resulting in extensive knowledge about health‐care systems, child symptoms, treatment processes and more. As a result, parents may draw from very different experiences, present unique skills and have specific needs—for example, psychosocial supports due to the impact of long‐term conditions on parenting—when compared with other populations.^11^, ^12^, ^13^ How to optimally engage parents in research therefore requires further attention.

Although the concept is known by many names, this article focuses upon engaging parents as “co‐researchers”, stemming from the definition of user involvement employed by INVOLVE, a UK government‐funded advisory group that supports active public involvement in health and social care research. The term refers to the act of carrying out research *with/by* health service users and family members who are not professional researchers, instead of *to/about/for* them.^7^ When engaged as co‐researchers, family members retain an active role and significant control over the course of the research, as well as a collaborative and interdependent relationship with the professional researchers.^14^, ^15^, ^16^

Engaging parents as co‐researchers recognizes them as experts with unique experiences and knowledge to contribute. This is believed to increase the quality and relevance of the research,^7^, ^8^ ensure acceptable and appropriate research designs,^6^, ^7^, ^8^, ^9^ and result in more credible and relevant outcomes.^10^, ^17^ Engaging parents as co‐researchers also aligns with widespread democratic and ethical principles by allowing those affected by a health issue to influence and have a voice in the research conducted regarding such health issues and conditions.^7^, ^9^

However, despite the increasing interest in engaging parents as co‐researchers,^16^, ^18^ explicit guidance on the process—how to actually do it—remains sparse.^10^ Therefore, the objectives of this scoping review were to (i) synthesize what is known about engaging parents as co‐researchers in health research; (ii) identify the potential benefits and challenges of engaging parent co‐researchers; and (iii) map existing evidence and identify gaps in the literature. To our knowledge, this review will be the first to conduct a rigorous synthesis in this area and is the first step to developing best practices in parent co‐researcher engagement.

## 2 Methods

### 2.1 Methodology

We utilized scoping review methodology as outlined by Arksey and O'Malley,^19^ and updated by others.^20^, ^21^ Designed to identify and map relevant literature, scoping reviews are ideal to investigate the breadth and depth of an emerging field of evidence where the literature is too heterogeneous to conduct a systematic review.^19^, ^20^, ^21^ As such, scoping review methodology presents an ideal method for mapping, extracting and summarizing an unclear body of evidence^21^ (such as engaging parents as co‐researchers), in a potentially comprehensive manner.^20^

### 2.2 Steering committee

Scoping reviews may refer to stakeholder consultation for feedback during any or many points in the course of the study.^21^ A steering committee composed of family‐centred care specialists (who are also parents of children with disabilities), researchers and health‐care managers collaborated throughout the course of our review. All members held an active role and were engaged throughout the course of the study, for example formulating research questions, priorities and inclusion and exclusion criteria, and providing input on article inclusion, potential resources and dissemination decisions.

### 2.3 Search strategy

We conducted a comprehensive literature search across four electronic databases (CINAHL, Medline, PsychInfo and Embase) with the support of an experienced academic librarian. The search strategy for each database comprised a combination of terms representing three concepts: (i) co‐researchers and research engagement (e.g. family engagement, parent involvement), (ii) the population engaged (e.g. parent, family) and (iii) the field of focus (e.g. research, biomedical research). The search was limited to articles published from 2005 to 2015, and in English. See Appendix 1 for a sample search strategy. Hand‐searching was also conducted with reference lists of included articles, as well as selected relevant bibliographies.^22^, ^23^ Several sources of grey literature were reviewed (listed in Appendix 2). Content experts on our steering group were also consulted for potential resources and sources of grey literature. A full list of search strategies, subject headings and keywords can be obtained from the last author.

### 2.4 Inclusion/exclusion criteria

To be included in the review, articles had to (i) focus on engaging parents/guardians (herein parents) in the process and/or design of research; (ii) describe the process and/or benefits of engaging parents in conducting research; and (iii) be related to health care. Exclusion criteria included the following: (i) parents were solely research subjects/participants; (ii) research focused solely on mental health, sexual health or oral health, given the specialized nature of these areas that warrant a separate review. Articles were also excluded if they engaged solely first‐time parents immediately post‐partum due to lack of experience in the parental role. Additionally, articles that provided insufficient data to complete 50% of the key fields in our data extraction table were excluded.

### 2.5 Data selection and extraction

Articles were initially screened using the inclusion and exclusion criteria via title and abstract. One reviewer (AS) screened all abstracts (n=8994), and a second researcher reviewed approximately 100 abstracts to calculate inter‐rater reliability. Conflicts were resolved by consensus, and a third reviewer (ACM) was brought in to review and settle further discrepancies. As inter‐rater reliability was high, author AS continued as the primary reviewer, retrieving and screening full text articles. Using the inclusion and exclusion criteria listed above, the primary reviewer (AS) was able to reduce all search results to the final included articles. All full text articles to be included were reviewed by the steering committee for relevance then charted in an electronic database created with Microsoft Excel via an iterative process concurrent with data extraction.^21^ The final table was checked by ACM for accuracy.

### 2.6 Summation, collation and synthesis

To map the available literature and identify significant gaps,^19^ an initial numerical summary was completed to provide an overview of included studies’ characteristics, such as the types of research design, geographical location, year of publication and study populations. Themes drawn from the papers that were relevant to the objectives were identified from the extracted data, grouped and discussed by the steering committee.

### 2.7 Quality assessment

While study inclusion is not determined by quality in scoping reviews,^19^, ^21^ assessing research quality can be useful when mapping the extent and nature of current literature. Quality assessment of empirical studies in the review was undertaken using Sirriyeh et al.'s^24^ quality assessment criteria. Designed for use with both qualitative and quantitative studies, the tool has 16 items, 14 for qualitative studies and 14 for quantitative studies. All 16 items are applicable where mixed methods have been used. Items are scored from zero to three, using a structured rubric delineating each score. The final quality score is expressed as a percentage (i.e. the raw score divided by either 14 or 16, depending upon the study design). Higher scores represent better quality.^24^

## 3 Results

**Figure 1:**
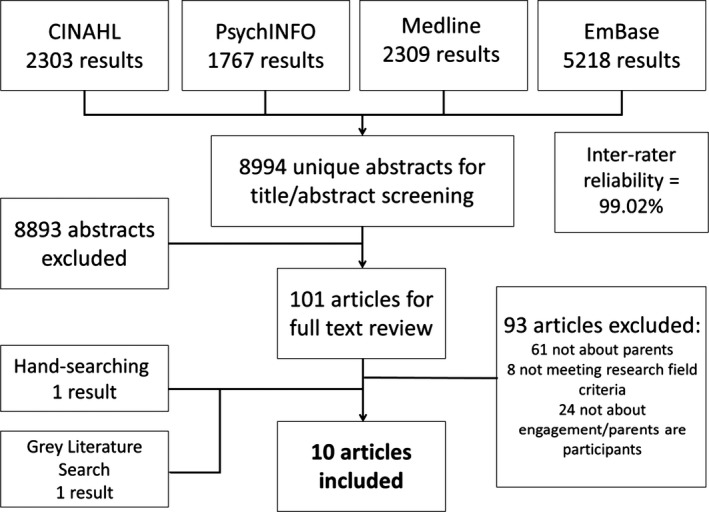
Search results and process

Our initial search yielded over 11 000 articles; 8994 were retained after removing duplicates with reference management software. Nine articles were included in the review. Inter‐rater reliability in the abstract screening phase averaged 99.02%. After hand‐searching and grey literature review, an additional report was identified, resulting in a total of 10 documents (herein referred to as “articles” for ease) that matched our inclusion criteria (Table 1). A full overview of the search process can be found in Fig. 1.

**Table 1:** Included studies

Author/year/country	Study design/Setting	Quality score	Research project	Co‐researcher group characteristics	Terminology used to describe engagement	Evaluation of impact	Benefits	Challenges
Blackburn et al.^25^ 2010, UK	Report (Grey Literature/Unspecified)	N/A	Study on postural care and support of children with complex disability in schools	Large group of parents in planning stage, two parents in steering committee; recruited via existing relationships	“Patient/public/user involvement”	Feedback and opinions of parent co‐researchers	More culturally responsive and sustainable intervention Increased # responses (parent co‐researchers provided momentum) Increased quality of results and rigour of research Increased reach in dissemination Parental empowerment	Inconsistency in expertise and motivations between parent co‐researchers and researchers (conflicts in priorities, lack of awareness of research unpredictability) Additional researchers required to effectively engage parent co‐researchers Increased cost (more time needed) Inconsistent experiences and opinions between parent co‐researchers (conflicting input)
Foster & Young^31^ 2015, UK	Case study/ Research facility	33.3%	Development of a large‐scale survey to obtain parental attitudes on sharing of neonatal health data for research purposes	11 Parents previously with a baby in neonatal care; varied age and socio‐economic status; predominantly female and white; recruited via advertisements	“Participatory research”, “Patient/public/user involvement”	Professional researcher analysis of proceedings	More culturally responsive and sustainable intervention More expertise contributed to greater research rigour Parent co‐researchers more likely to take emancipatory action	Concerns of tokenistic parental engagement Additional researchers required to effectively engage parent co‐researchers Inconsistent experiences and opinions between parent co‐researchers (providing conflicting input)
Greenmills et al.^34^ 2013, USA	Descriptive/ Community programme	N/A	Development of a childhood obesity prevention initiative	Parents of children involved in Head Start programmes; from disadvantaged areas	“Co‐researcher”	Results of research project compared to similar intervention without parent co‐researchers	More culturally responsive and sustainable intervention	None stated
Jurkowski et al.^32^ 2013, USA	Case study/ Community programme	42.9%	Development of a childhood obesity prevention initiative	13 parents/grandparents of children in the community with consistent attendance by 10; low‐income; 90% female; recruited via contact with community partners	“Community‐based participatory research”, “Parent engagement”, “Co‐researcher”	Parent interviews, anecdotes, evaluation surveys	Parent co‐researchers contributed more expertise, led to more parent co‐researchers More culturally responsive and sustainable intervention Parental empowerment and chance of emancipatory action	Unclear roles Inconsistent education levels and research expertise between parent co‐researchers and researchers
Rowe^26^ 2006, UK	Case study/ Community programme	45.2%	Development of a survey to evaluate a community child health programme	16 mothers; from disadvantaged areas; recruited via advertised posts and existing relationships	“Parent researcher”, “Lay researcher”	Parent anecdotes, evaluation responses, and research diary data with professional researcher analysis of proceedings	More culturally responsive and sustainable intervention Increased # responses Increased credibility in dissemination Parental empowerment and chance of emancipatory action	Struggles with power differential and unclear role distinction Additional researchers required to effectively engage parent co‐researchers Inconsistent expertise, motivations and expectations between parent co‐researchers and researchers Inconsistent experiences and opinions between parent co‐researchers (providing conflicting input) Increased costs Parent co‐researchers required additional skills and training to be engaged
Staniszewska et al.^27^ 2007, UK	Case study/ Community	31.0%	Development of a research bid to explore parental experience of having a pre‐term infant	Parents of premature babies involved in a pre‐term support group; recruited via existing relationships	“Patient/public/user involvement”	N/A	More culturally responsive and sustainable intervention Increased quantity of results (parent co‐researchers provide momentum for more research) Increased credibility and reach in dissemination Parental empowerment and chance of emancipatory action	Struggles with power differential and unclear role distinction Increased cost (time and money) Inconsistency in expertise between parent co‐researchers and researchers (lack of awareness of research unpredictability causing frustration) Unpredictability in parental life causing timing constraints, conflicts
Stuttaford & Coe^28^ 2007, UK	Descriptive/ Community programme	N/A	Evaluation of a community child health programme	Parents involved in community Sure Start programmes; from disadvantaged areas	“Participatory research”	Parent feedback	Increased # responses Parental empowerment and chance of emancipatory action	Parent co‐researchers require additional skills and training to be engaged
Uding et al.^29^ 2007, USA	Case study/ Research facility	38.1%	Development of a psycho‐educational programme for parents/primary caregivers of children with special health care needs	27 total parents of children with special health care needs; diverse ethnicities; recruited via existing relationships and job advertisements	“Participatory research”, “Parent co‐investigator”	N/A	Parent co‐researchers led to more participants More culturally responsive and sustainable intervention Increased quality of results and rigour of research Increased credibility in dissemination	Unclear role distinctions Inconsistent experiences and opinions between parent co‐researchers (conflicting input) Inconsistent education levels and research expertise between parent co‐researchers and researchers (parent co‐researchers unaware of research unpredictability) Timing constraints and conflicts Increased costs
Uding et al.^33^ 2009, USA	Descriptive/ Research facility	N/A	Development of a psycho‐educational programme for parents/primary caregivers of children with special health‐care needs	11 parents of children with long‐term conditions; recruited via existing relationships and community contacts	N/A	N/A	Parent co‐researchers led to more participants Greater patient/public/user involvement More culturally responsive and sustainable intervention Increased quality of results and rigour of research Increased reach in dissemination	Need for more skilled researchers to effectively engage parent co‐researchers Increased cost (more time)
Walmsley & Mannan^30^ 2009, Ireland	Case study/ Unspecified	45.2%	Qualitative study to explore experience of families of people with intellectual disability	10–14 family members (focus on parents) of children with intellectual disability; recruited via advertisements and existing relationships	“Participatory action research”	Co‐researcher interviews with professional researcher analysis of proceedings	More culturally responsive and sustainable intervention Increased # responses Increased credibility in dissemination Parental empowerment and chance of emancipatory action	Parent co‐researcher lack of experience with research process (frustrated with lack of immediate action from findings)

### 3.1 Article characteristics

The articles were published in the United Kingdom (n=5), United States (n=4) and the Republic of Ireland (n=1). The majority (n=6) of articles were published in or after 2009. Little detail was provided regarding the characteristics of the parent co‐researchers. Selected characteristics regarding the population and research settings among included articles can be found in Table 2, which highlights patterns in demographic data, number of parent co‐researchers and the settings, when specified.

**Table 2:** Population characteristics and research settings

	n
Characteristics of parent co‐researchers engaged	
Mostly or only women	3
Low income/areas of disadvantage	3
Ethno‐cultural diversity	3
Number of parent co‐researchers engaged	
<10	1
10–20	5
>20	1
Unspecified	3
Research setting	
Community	5
Research Institute	3

### 3.2 Terminology used

A wide range of terms were used across studies to describe co‐researcher engagement. Most common were as follows: “patient/public/user involvement” (n=3) and “participatory research” (n=3), followed by “co‐researcher” (n=2). The terms “parent co‐investigator,” “parent researcher,” “lay researcher,” “participatory action research,” “community‐based participatory research” and “community‐based participatory research” were each used in a single article.

### 3.3 Design and quality

Included articles were classified as case studies (n=6), descriptive articles (n=3) and reports (n=1). Healthy lifestyles and obesity research was the most common field of study (n=4), followed by disability and long‐term disease (n=3), and infant health (n=3). The majority of articles engaged parent co‐researchers in research informing programme development, implementation and/or evaluation (n=6). Quality assessment was applicable to six empirical articles, with a mean score of 39.2%. The maximum score was 45.2%, with a lowest score of 31.0%.

### 3.4 Methods and types of engagement

#### 3.4.1 Recruitment and group composition

The majority of the articles (n=6)^25^, ^26^, ^27^, ^28^, ^29^, ^30^ made use of existing relationships (e.g. contacting members of a hospital advisory group or community programme) to identify potential parent co‐researchers. Other recruitment methods included the use of advertisements or job postings,^26^, ^29^, ^30^, ^31^ or leveraging key community contacts such as nurses or teachers.^29^, ^32^ Most researchers engaged parent co‐researchers continuously through the course of the project via either small groups of the same one to four parents,^25^, ^29^, ^30^ or large groups with fluctuating member composition.^26^, ^28^, ^31^, ^32^ Other researchers made use of large focus groups of parents for input at intermittent points through the course of the research process.^25^, ^29^, ^30^

#### 3.4.2 Research processes

Studies involved parent co‐researchers in different aspects of the research process (Table 3 provides an overview). Parent co‐researchers were reported as having been engaged in developing research questions, aims and objectives and setting research priorities.^25^, ^27^, ^31^, ^32^ Less commonly, they contributed to project conceptualization or assisted in grant writing.^27^, ^29^ Three articles reported engaging parent co‐researchers in designing qualitative studies aiming to explore parental attitudes and experiences in relation to their child's health.^27^, ^30^, ^31^ Parent co‐researchers often played a key role in developing research interventions by, for example, developing programme curriculums,^29^, ^32^ critiquing the design, language and content of an intervention, as well as logistics, for example participant compensation.^27^, ^29^, ^33^ Parent co‐researchers also contributed to the development and revision of data collection instruments such as outcome measures,^29^, ^30^, ^32^ interview schedules,^27^, ^28^ focus group questions^30^, ^32^ and questionnaires.^26^, ^31^, ^34^ They were also engaged in implementing interventions and collecting data, for example, by facilitating focus groups^29^, ^30^, ^32^ or conducting interviews.^26^, ^28^

**Table 3:** Areas in the research process where parent co‐researchers were engaged

Author	Year	Stages entailing co‐researcher engagement									
		Research planning		Study design and methodology				Results analysis		Dissemination	
		Project concept ualization	Developing research questions	Developing recruitment methods	Developing intervention	Developing outcome measures	Facilitating intervention	Data analysis	Data entry	Developing knowledge translation plans	Participating in dissemination
Blackburn et al.^25^	2010		✔					✔	✔	✔	✔
Foster & Young^31^	2015		✔	✔	✔						
Greenmills et al.^34^	2013				✔			✔			
Jurkowski et al.^32^	2013		✔		✔	✔	✔	✔		✔	✔
Rowe^26^	2006				✔		✔	✔			✔
Staniszewska et al.^27^	2007	✔	✔		✔					✔	
Stuttaford & Coe^28^	2007				✔		✔	✔			
Uding et al.^29^	2007	✔		✔	✔	✔	✔	✔		✔	✔
Uding et al.^33^	2009				✔						
Walmsley & Mannan^30^	2009				✔	✔	✔				✔

#### 3.4.3 Data analysis and dissemination

After the collection of data, several articles stated that parent co‐researchers performed data entry and analysis^25^, ^26^, ^28^, ^29^, ^32^, ^34^ and formulated recommendations.^25^, ^26^, ^29^ Parent co‐researchers developed plans for dissemination^25^, ^27^, ^29^, ^32^ and took part in disseminating results,^29^, ^30^ for example, by writing abstracts, presentations, reports and posters,^25^, ^26^, ^32^ or participating in conference presentations.^25^, ^26^, ^29^, ^31^, ^32^

#### 3.4.4 Facilitators/enablers

Several articles reported specific factors that facilitated parent engagement. Offering childcare^26^, ^29^, ^32^ and meals^25^, ^32^ was reported to be beneficial, as well as payment in the form of gift cards or monetary reimbursement.^26^, ^29^, ^32^ Two articles employed the parent co‐researchers as volunteers.^25^, ^31^ Five articles described providing some form of training to parent co‐researchers, although in most cases this training was to facilitate the specific tasks the parent co‐researchers were performing, for example how to conduct an interview or deliver an intervention, rather than a broader research curriculum.^26^, ^28^, ^29^, ^30^, ^32^

### 3.5 Benefits of parental engagement

#### 3.5.1 Evaluating success of parental engagement

When specified, articles reported using anecdotal comments to evaluate the result of parent co‐researcher involvement,^25^, ^28^ survey responses,^26^, ^32^ individual interviews^30^, ^32^ and research diary data.^26^ Three of the articles reported that the researchers analysed the proceedings of the research project to determine results via analysis of recordings and self‐reflections to identify key themes.^26^, ^30^, ^31^ In one study, the impact of involving parent co‐researchers in making messages more relevant and meaningful to other parents was assessed by comparing the rates of parents recalling having seen the campaign messages to those “typically observed in child health promotion campaigns targeting parents through mass media.”^34^ ^p31^ Three articles did not mention the use of any form of outcome evaluation.^27^, ^29^, ^33^

#### 3.5.2 Benefits for researchers

Nine of the 10 articles concluded that parent engagement led to more sustainable and population‐appropriate interventions.^25^, ^26^, ^27^, ^29^, ^30^, ^31^, ^32^, ^33^, ^34^ The resulting research was reported to be more meaningful and culturally/socio‐economically appropriate, identifying issues and details that researchers may not have been initially aware of. For example, parent co‐researchers helped to optimize intervention timing and the location of data collection to accommodate the needs of participants, thereby maximizing participant involvement and attendance, and thus increasing the amount of data gathered (e.g. response rates on a survey). In addition, four articles made note of the passion and enthusiasm exhibited by parent co‐researchers, which motivated researchers and fuelled momentum and ideas for future research.^25^, ^26^, ^27^, ^30^

Four articles identified wide‐ranging experiences between parent co‐researchers, in both their experiences of caring for children with differing needs, and in how their lifestyles differed.^25^, ^29^, ^31^, ^33^ These factors were thought to have contributed to an increased pool of expertise and opinions, leading to greater rigour in decision making and overall increased quality of results. Parent co‐researcher involvement was also thought to have increased the reach of dissemination,^25^, ^27^, ^33^ as well as the credibility of the research with patient/parent groups and professionals.^26^, ^27^, ^29^, ^30^

#### 3.5.3 Benefits for parents

Several articles reported parent co‐researcher empowerment resulting from engagement in the research, due to increased confidence and research skills,^26^, ^30^, ^32^ as well as obtaining a sense of control over health service involvement.^25^, ^27^ Engaging parent co‐researchers also increased their awareness of health issues and increased the likelihood of making changes in the area of focus in the future,^27^, ^28^, ^30^ for example being involved in community programmes after completion of the research project.^26^, ^28^, ^31^, ^32^

### 3.6 Challenges to engagement

#### 3.6.1 Challenges for researchers

A common issue reported by many articles was the perceived increase in resources required to engage parent co‐researchers. For example, extra time was reported as necessary for building relationships with the parent co‐researchers and to resolve any conflicts, as well as to incorporate their feedback.^25^, ^26^, ^29^, ^33^ Increased monetary costs could also be incurred as a result of more time spent on the project, and the need to compensate and support the parent co‐researchers.

A further challenge arose from disconnects between parent co‐researcher and researcher foci of interest, as researchers were perceived to be driven by literature gaps and funding agency directives, which did not always align with the concerns of the parent co‐researchers.^25^, ^26^, ^27^ Two articles addressed the risk of a power imbalance between researchers and parent co‐researchers, reporting that researchers should take a facilitative, rather than leading role, as per the traditional research paradigm.^26^, ^27^ For example, researchers would often lead the actual data analysis and/or relay information to parents in a synthesized and lay manner.^25^, ^26^, ^27^, ^30^, ^32^ Several studies raised concerns that the true power still remained with the researchers, potentially resulting in tokenistic parental involvement.^26^, ^31^ As a result, a number of articles addressed the need for researchers to have specialized skills to truly engage parents as equals.^25^, ^26^, ^27^, ^31^, ^33^

#### 3.6.2 Challenges for parents

Differences among parent co‐researchers, and/ or between parent co‐researchers and researchers appeared to create several challenges. Inconsistent educational levels and research expertise were reported to result in parent co‐researcher disappointment, frustration and powerlessness due to a lack of awareness of certain research logistics, for example the inherent unpredictability of methods and results in research,^25^, ^26^, ^27^, ^29^ as well as the lack of immediate action as a result of their contributions.^26^, ^27^, ^30^ Two articles also outlined potential parent disengagement resulting from unclear roles and task distinctions between the researchers and parent co‐researchers.^29^, ^32^ Parent co‐researchers themselves could represent a wide range of experiences and opinions, potentially causing conflict and a lack of consensus on the resulting decisions.^25^, ^26^, ^29^, ^31^

## 4 Discussion

We found limited literature addressing the involvement of parents as co‐researchers in research, despite the move towards greater patient involvement elsewhere in health care. The studies included in this scoping review demonstrated variability in how and when they engaged parents as co‐researchers. Most of the studies involved parents in the development of interventions, their implementation and/or evaluation (n=6), or qualitative studies (n=3), indicating a need for greater parent engagement in a broader range of study designs and across the many stages of research projects. Parent involvement was notably largely absent from the conceptualization of studies, demonstrating that professional researchers generally engage parents in research after the study focus has been finalized. There may be many reasons for this, not least the opportunistic nature and narrow focus of many research funding opportunities. Future research may usefully examine earlier engagement of parent co‐researchers to further integrate them into having greater influence and autonomy in health research. Greater collaboration between researchers and parent co‐researchers has potential benefits for all involved, such as enhanced relevance to the target population, increased quality of research processes and clearer directions for future research.

It is important to note that a substantial body of literature already exists on the topic of patient engagement, including descriptive articles and frameworks,^5^, ^6^, ^9^, ^15^, ^35^ case studies,^14^, ^17^, ^36^, ^37^, ^38^ reports^2^, ^7^, ^16^, ^39^ and reviews.^8^, ^10^, ^18^, ^40^, ^41^, ^42^ This larger body of evidence echoes much of the literature we identified for parent co‐researcher engagement specifically, such as a lack of early engagement, poor quality of evidence and heterogeneity in approaches to engagement. The benefits and challenges that have been reported with other populations are also similar to our findings. However, involving parents as a proxy for their child's voice, as well as leveraging their expertise, has drawn less research attention. Although there are similarities between our findings and those concerned with other patient populations involved in research, the emerging evidence we identified suggests several issues that should be considered when engaging parents as co‐researchers. First, timing was a common issue—parents caring for children with long‐term illnesses may deal with employment and financial issues in addition to their caring duties.^11^, ^13^ Parent co‐researchers reported having little free time, which created a need for flexibility and certain facilitators (childcare, timing of meetings, meals, payment). Second, a noted issue among parents of children with health‐care issues, particularly those caring for children with long‐term conditions, is a perceived lack of control.^12^, ^43^, ^44^ Therefore, the empowerment and fulfilment resulting from being engaged in research may be particularly valuable. Third, parents feasibly have a certain sense of vigilance related to health care—a commitment through the life of their children, and a motivation to learn and do all that they can for their child^13^, ^44^, ^45^—which may contribute to the passion and drive that researchers felt from their parent co‐researchers.

As such, it is important for future research to further examine parent co‐researcher engagement in other fields of health research, particularly where well children are not involved and cannot advocate for themselves—such is the case with long‐term conditions, rehabilitation, acquired injury and many more. This is particularly true when considering that much of the available literature on parent co‐researcher engagement occurs in the context of public health and health promotion.

Even where researchers are willing to engage with parent co‐researchers, we did not identify any clear guidelines on how to do so. However, the results of this scoping review provide some indication of practices that may be beneficial and can serve as the basis for subsequent research endeavours to inform best practices when researchers are engaging parents as co‐researchers (see Box 1 for an overview).

Box 1 **Recommendations for engaging parents in research**

1

- Engage parents as early as possible to build relationships to maximize their impact^7^, ^8^, ^17^, ^25^, ^27^, ^29^, ^32^, ^36^
- Provide support, encouragement and recognition to parents for their contributions, recognize parents as experts and equals,^27^, ^28^, ^31^ be responsive to parents’ lifestyles.^25^, ^29^ Some of the topics discussed are very personal to the parents and can elicit emotional responses^7^, ^8^, ^15^, ^25^, ^26^, ^27^
- Be clear on roles—outline the differences in duties and expectations between the co‐researchers and professional researchers^7^, ^8^, ^9^, ^15^, ^29^, ^32^
- Provide relevant training to parent co‐researchers^26^, ^28^, ^29^, ^30^, ^32^
- Have a trusting and positive work environment by providing structural supports—for example meet in convenient places, provide monetary incentives/ reimbursements, provide food and childcare and create group guidelines; all help show commitment to parents^15^, ^26^, ^29^, ^32^
- Plan for unpredictability—have backup plans, conflict resolution strategies, make the research process transparent and ensure parents are aware of everything from the start, including the inherent unpredictability of research^25^, ^27^, ^29^, ^30^, ^31^

One key challenge when conducting this scoping review was that the concept of engaging patients and families has been referred in the literature using diverse terminology. Among the articles included in this review, no single term had more than three articles using it, with the majority of terms having only one instance. Within the broader literature, Turnbull et al. have identified the terms “participatory research,” “action research,” “participatory action research,” “constituency‐oriented research and dissemination”, “emancipatory research”, “empowerment research”, and “discovery research”.^9^ Additionally, the terms “patient/public involvement,” “patient/service user engagement,” “lay involvement” and “public consultation” emerged in our searches. This heterogeneity in terminology causes multiple challenges. For one, capturing all the available literature on the topic is challenging because studies are classified so uniquely. To identify relevant studies, we had to conduct broad searches, resulting in large numbers of studies to screen, requiring time and resources. Definitions were also rarely provided, taking additional time to carefully determine the level of parental involvement in the research project. The terms “participatory action research,” “action research” and “emancipatory research” are considered a qualitative research method engaging community members and settings,^46^ but are conceptually distinct from parental co‐researcher engagement that we are examining (which is an element that can be integrated into any project).^7^ However, some papers conflated the terms and used “participatory action research” alongside elements of co‐researcher engagement. It was therefore challenging to distinguish between different approaches and reinforces our call for standardized terminology to be used. We therefore recommend having a universally defined set of terminology to avoid such challenges and advance future research. “Patient engagement” has become commonly used, including with the U.S. Food and Drug Administration^47^ and the Government of Canada,^48^ to describe the involvement of patients and families in affecting health care beyond simply at the point of care, such as in research, policy setting and public service campaigns.^8^, ^10^, ^49^ However, as the term is still somewhat ambiguous, “co‐researcher” may be appropriate as an additional, more categorical term to refer to patient engagement in research specifically.

### 4.1 Considerations

The research included in this study was primarily conducted in the United Kingdom and United States, reducing the generalizability of their recommendations (where stated). There is therefore a clear lack of evidence across different countries, which is potentially important due to differences in health‐care delivery and research settings. Due to the emerging state of the evidence, the full impact of parent co‐researchers on the research field is as yet unclear. However, as parents remain proxies for the voice of children with severe disabilities for most or the entirety of their lives, there are clearly potential benefits to exploring further how best to engage them in research. Although scoping reviews are not designed to give weight to studies’ findings based on quality assessment,^19^, ^21^ the diversity in the quality of studies that we located must be considered when interpreting our findings. For example, the six empirical studies that underwent quality assessment were of relatively low quality, with the highest quality score being 45.2%, and the lowest being 31.0%. One reason was the lack of any specific framework, resulting in inconsistencies and lack of structure among the studies. There was a notable lack of demographic data in the included articles, such as the age of parent co‐researchers’ children, and the socio‐economic status, ethnicity and gender of the parent co‐researchers. More detailed reporting of this information would allow greater understanding of how they impact upon parental engagement. Many of the studies were lacking rigour in outcome measures and analysis. Three articles did not report any form of evaluation of impact, while the majority of those that did (n=6) utilized relatively subjective forms of evaluation, such as co‐researcher and professional researcher observations and anecdotes.

Our searches were also limited to CINAHL, Medline, PsychINFO and Embase databases between 2005 and 2015 and the two bibliographies and 11 websites (including online grey literature databases) hand‐searched, which may have limited our findings. Although we tried to be inclusive, due to the heterogeneity of terminology discussed earlier, some search terms may have been missed in the search strategy. The dates were restricted for maximal use of the available resources and the lack of relevant studies identified before 2005 in an initial scan. We also chose to exclude research in mental, sexual or oral health, given the specific nature of the topics. Despite these considerations, the detailed overview of the literature provided by this review identified many factors—including pragmatic, philosophical and ethical—to consider when engaging parents as co‐researchers. We also minimized the possibility of missing papers as much as possible by advancing any paper that was unclear regarding eligibility in the abstract screening phase (e.g. could possibly have engaged parents), for full text screening. This is one reason why we screened 101 full text articles, plus additional hand‐searching, but only yielded 10 final articles.

## 5 Conclusions

The current evidence suggests that engaging parents as co‐researchers brings both benefits and challenges, but can potentially enhance research that is acceptable and relevant for the population it is intended to serve. Despite this, there is insufficient high‐quality research to create evidence‐based best practice guidelines for how to engage parents as co‐ researchers at this time. However, our scoping review provides a synthesis of available evidence on this under‐researched topic and can guide future research focusing upon structured frameworks and rigorous approaches to engaging parents equitably in health research.

## Funding Sources

The study was funded by the Ward Family Summer Student Program and the Bloorview Research Institute.

## Conflicts of Interest

No conflict of interests to declare.
