# Supplementary material for: How and why should we engage parents as co‐researchers in health research? A scoping review of current practices
Source: Health Expect. 2016 Aug 12;20(4):543–54. doi: 10.1111/hex.12490 (PMC5513005; doi:10.1111/hex.12490)
Supplement: Supplementary file 1 [file HEX-20-543-s001.docx]

**Supplemental file 1. Sample search strategy.**

| 1 | family-led.mp. |
| --- | --- |
| 2 | (parent* adj3 engag*).mp. |
| 3 | (parent* adj3 involv*).mp. |
| 4 | (parent* adj3 consult*).mp. |
| 5 | (parent* adj3 participa*).mp. |
| 6 | parent-led.mp. |
| 7 | (consumer* adj3 engag*).mp. |
| 8 | (consumer* adj3 involv*).mp. |
| 9 | (consumer* adj3 consult*).mp. |
| 10 | (consumer* adj3 participa*).mp. |
| 11 | consumer-led.mp. |
| 12 | (user* adj engag*).mp. |
| 13 | (user* adj3 involv*).mp. |
| 14 | (user* adj3 consult*).mp. |
| 15 | (user* adj3 participa*).mp. |
| 16 | user-led.mp. |
| 17 | co-researcher*.mp. |
| 18 | coresearcher*.mp. |
| 19 | (participatory adj2 research).mp. |
| 20 | patient participation/ |
| 21 | (parent* adj3 research*).mp. |
| 22 | (family adj3 engag*).mp. |
| 23 | (family adj3 involv*).mp. |
| 24 | (family adj3 consult*).mp. |
| 25 | (family adj3 participa*).mp. |
| 26 | (families adj3 engag*).mp. |
| 27 | (families adj3 involv*).mp. |
| 28 | (families adj3 consult*).mp. |
| 29 | (families adj3 participa*).mp. |
| 30 | co-investigat*.mp. |
| 31 | coinvestigat*.mp. |
| 32 | 1 or 2 or 3 or 4 or 5 or 6 or 7 or 8 or 9 or 10 or 11 or 12 or 13 or 14 or 15 or 16 or 17 or 18 or 19 or 20 or 21 or 22 or 23 or 24 or 25 or 26 or 27 or 28 or 29 or 30 |
| 33 | 1 or 2 or 3 or 4 or 5 or 6 or 7 or 8 or 9 or 10 or 11 or 12 or 13 or 14 or 15 or 16 or 17 or 18 or 19 or 20 or 21 or 22 or 23 or 24 or 25 or 26 or 27 or 28 or 29 or 30 or 31 |
| 34 | parent*.mp. |
| 35 | caregiver*.mp. |
| 36 | carer*.mp. |
| 37 | exp parent/ |
| 38 | caregiver/ |
| 39 | family relation/ or family/ or exp child parent relation/ |
| 40 | families.mp. |
| 41 | family.mp. |
| 42 | 34 or 35 or 36 or 37 or 38 or 39 or 40 or 41 |
| 43 | (participatory adj3 research).mp. |
| 44 | (collaborati* adj3 research).mp. |
| 45 | action research/ or analytical research/ or applied research/ or basic research/ or behavioral research/ or medical research/ or clinical nursing research/ or clinical research/ or participatory research/ or comparative effectiveness/ or descriptive research/ or ethnographic research/ or ethnonursing research/ or evaluation research/ or exploratory research/ or nutritional science/ or genetics/ or gerontological research/ or health services research/ or human factors research/ or interdisciplinary research/ or mental health research/ or methodology/ or nursing administration research/ or nursing evaluation research/ or nursing methodology research/ or nursing research/ or outcomes research/ or pediatrics/ or "peer review"/ or phenomenology/ or population research/ or qualitative research/ or health care quality/ or rehabilitation research/ or research/ or research ethics/ or research priority/ or research subject/ or translational research/ or cancer research/ |
| 46 | research*.ti. |
| 47 | 43 or 44 or 45 or 46 |
| 48 | 43 or 44 or 45 or 46 |
| 49 | 33 and 42 and 47 |
| 50 | limit 49 to yr="1990 -Current" |
| 51 | limit 50 to yr="1995 -Current" |
| 52 | limit 49 to yr="2000 -Current" |
| 53 | limit 51 to yr="2005 -Current" |
| 54 | limit 53 to english language |
| **55** | **limit 54 to human** |

**Supplemental file 2. Grey literature sources.**

| Grey Literature Sources | |
| --- | --- |
| INVOLVE  James Lind Alliance  Peninsula Cerebra Research Unit  Canadian Foundation for Healthcare Improvement  Institute for Patient and Family Centered Care  Open Grey | Health Canada  Beach Center on Disability at the University of Kansas  Patient Centered Outcomes Research Unit  Lucille Packard Children’s Institute  Canadian Institute for Health Information |
